# An Instrumented Glove-Controlled Portable Hand-Exoskeleton for Bilateral Hand Rehabilitation

**DOI:** 10.3390/bios11120495

**Published:** 2021-12-03

**Authors:** Shih-Hung Yang, Chia-Lin Koh, Chun-Hang Hsu, Po-Chuan Chen, Jia-Wei Chen, Yu-Hao Lan, Yi Yang, Yi-De Lin, Chun-Hung Wu, Hsien-Kuang Liu, Yu-Chun Lo, Guan-Tze Liu, Chao-Hung Kuo, You-Yin Chen

**Affiliations:** 1 Department of Mechanical Engineering, National Cheng Kung University, Tainan 70101, Taiwan; vssyang@gs.ncku.edu.tw; 2 Department of Occupational Therapy, College of Medicine, National Cheng Kung University, Tainan 70101, Taiwan; clkoh@gs.ncku.edu.tw; 3 Department of Electrical Engineering, National Chung Cheng University, Taichung 40227, Taiwan; g08415007@ccu.edu.tw; 4 School of Electrical and Computer Engineering, Georgia Institute of Technology, Atlanta, GA 30332, USA; pchen353@gatech.edu; 5 Department of Biomedical Engineering, National Yang Ming Chiao Tung University, Taipei 11221, Taiwan; sk413025@gmail.com (J.-W.C.); leolan9534766@gmail.com (Y.-H.L.); ianyang01@gmail.com (Y.Y.); chaohungk@gmail.com (C.-H.K.); 6 Department of Mechanical and Computer-Aided Engineering, Feng Chia University, Taichung 40724, Taiwan; led357829@gmail.com (Y.-D.L.); as756954@gmail.com (C.-H.W.); hkliu@fcu.edu.tw (H.-K.L.); 7 College of Medical Science and Technology, Taipei Medical University, Taipei 11031, Taiwan; aricalo@tmu.edu.tw; 8 Department of Medical Education, Taipei Veterans General Hospital, Taipei 112201, Taiwan; tze6587@gmail.com; 9 School of Medicine, National Yang Ming Chiao Tung University, Taipei 11221, Taiwan; 10 Department of Neurological Surgery, Neurological Institute, Taipei Veterans General Hospital, Taipei 11217, Taiwan; 11 Department of Neurological Surgery, University of Washington, Seattle, WA 98195-6470, USA

**Keywords:** hand exoskeleton, hand-sensing glove, bilateral hand training, virtual reality game

## Abstract

Effective bilateral hand training is desired in rehabilitation programs to restore hand function for people with unilateral hemiplegia, so that they can perform daily activities independently. However, owing to limited human resources, the hand function training available in current clinical settings is significantly less than the adequate amount needed to drive optimal neural reorganization. In this study, we designed a lightweight and portable hand exoskeleton with a hand-sensing glove for bilateral hand training and home-based rehabilitation. The hand-sensing glove measures the hand movement of the less-affected hand using a flex sensor. Thereafter, the affected hand is driven by the hand exoskeleton using the measured hand movements. Compared with the existing hand exoskeletons, our hand exoskeleton improves the flexible mechanism for the back of the hand for better wearing experience and the thumb mechanism to make the pinch gesture possible. We designed a virtual reality game to increase the willingness of repeated movement practice for rehabilitation. Our system not only facilitates bilateral hand training but also assists in activities of daily living. This system could be beneficial for patients with hemiplegia for starting correct and sufficient hand function training in the early stages to optimize their recovery.

## 1. Introduction

Hand function which requires dedicated finger flexion and extension movements is an important basis for executing activities of daily living, such as holding a glass of water, grabbing a fork, and holding a pen. Impaired hand function affects an individual’s ability to be independent [1]. Dyskinesias often occur in patients with stroke, spinal cord injury, neuromuscular disease, and amyotrophic lateral sclerosis. Brain injury-induced hand function impairment restricts patients from autonomously interacting with their surroundings, significantly reducing their quality of life [1,2]. These patients require rehabilitation in the hospital [3] and assistance from their families in their daily lives, resulting in high medical and care costs [4]. Therefore, restoration of hand function is a major treatment goal for patients with hemiparetic arm to optimize recovery outcomes [5].

Bilateral hand training, which is a rehabilitation treatment, is useful and promising [6] for patients with unilateral hemiparesis or hemiplegia [7]. Both hands are used symmetrically to perform a task, e.g., grasping an object. The bilateral hand training thus enhances performance of the affected hand. Studies suggest that bilateral arm movements activate additional brain circuits, such as the supplementary motor area and primary motor cortex across the hemispheres, which further benefits the motor recovery of the affected hand [8]. Using the less-affected arm facilitates the restoration of hand function in patients with unilateral hemiplegia. Therefore, effective bilateral hand training is desired in rehabilitation program to restore patients’ hand function, allowing them to perform daily activities independently.

A large number of repeated and correct movement practices are the three crucial elements of rehabilitation to restore the hand function in patients with unilateral hemiplegia. However, owing to limited human resources, the movement practice provided in current clinical settings is significantly less than the adequate amount needed to drive optimal neural reorganization [9]. The hand exoskeleton we designed has several integrated features to promote home-based rehabilitation to maximize patients’ opportunity for recovery, including low-cost, portability, safety, and self-assisted training. For patients with hemiplegia, motor training of the affected hand heavily relies on the physical guidance of therapists or caregivers to provide correct sensorimotor input to the limb without voluntary movement. Using the hand exoskeleton, patients with hemiplegia can carry out bilateral hand training at home or in clinics to increase the amount of correct repetitive practice to prompt neural repair for the affected hand function. In addition, the patients’ motivation for motor training could be increased by using a hand exoskeleton with virtual reality games. This system could be beneficial for patients with hemiplegia to start correct and sufficient hand function training in the early stages to optimize their recovery. Furthermore, the burden on caregivers and the medical costs can be reduced. 

## 2. Related Works

Automatic bilateral hand training requires sensors to measure movements of the less-affected hand and hand exoskeleton to actuate the affected hand. The hand exoskeleton usually provides a partial assistant force to drive finger flexion and extension [10]. Table 1 summarizes the state-of-the-art hand exoskeletons, including the number of fingers, number of motors, degree of freedom, and transmission mechanism. Most rehabilitations using hand exoskeletons are conducted in hospitals because the cost of the machine is too high to be popular in most families. Therefore, we developed a low-cost hand exoskeleton.

Conventional hand exoskeletons apply a motor (weighing approximately 240 g) to actuate the mechanism and equip the motor on the hand or forearm sides, leading to large physical loading. In et al. proposed a jointless mechanism for hand exoskeleton, which transmits force by steel wire ropes [11]. This mechanism is lightweight, weighing only 80 g. However, it is difficult to fit various hand sizes of individuals. Nycz et al. [12] improved the portability of the hand exoskeleton by introducing a sliding spring instead of wire ropes. The actuators are attached to the back of an individual to reduce the loading of the hand and arm. However, this hand exoskeleton lacks the thumb mechanism, making it unable to perform pinch, which requires the action of the index finger and thumb for various daily life activities. 

**Table 1:** Comparison of state-of-the-art hand exoskeletons. #: number of DoF, degree of freedom; Trans., transmission mechanism. *: the weight of the complete system includes the hand exoskeleton, hand-sensing glove, linear motors, and battery.

Study	# Fingers	# Motors	Weight (g)	DoF	Trans.
HandSOME [13]	2	0	-	1	Springs/links
HANDEXOS [14]	1	1	115	5	Steel wire ropes
Gloreha [15]	5	-	-	-	Steel wire ropes
Wege et al. [16]	5	5	-	20	Steel wire ropes
Ueki et al. [17]	5	11	-	18	Links
Rehabotics [18]	5	-	-	-	Steel wire ropes
Hand of Hope [19]	5	-	-	-	Links
In et al. [20]	0	0	80	1	Steel wire ropes
Tadano et al. [21]	5	-	-	10	Pneumatics
DiCicco et al. [22]	2	-	-	2	Pneumatics
KULEX [23]	2	1	-	1	Links
Nycz et al. [12]	4	4	113	4	Sliding springs
Yurkewich et al. [24]	5	2	-	-	Fishing wire tendons
Gasser et al. [25]	5	1	360	1	Wire
Li et al. [26]	5	6	500	6	Links
Ahmed et al. [27]	5	14	280	14	Links
Zhang et al. [28]	3	1	352	4	Tendon-actuated
Ours	5	5	1040 *	5	Sliding springs

The control command of the hand exoskeleton can be generated by the less-affected hand or intention of the user. Electromyography (EMG) provides an alternative way to present the intention of the user [19]. The EMG signal occurs before the real human movement, and it is related to the human joint torque. However, the EMG sensor is sensitive to neural properties and has limitations in practical applications. Moreover, EMG may not measure the user’s intention in patients with brain injury or spinal cord injury. In addition, muscle fiber extension and muscle stiffness sensors have been proposed to estimate a user’s intention of movement. However, they may be unstable under a few conditions. Bilateral hand training provides an alternative way to measure the movement of the less-affected hand and then drive the affected hand. However, the existing systems are expensive and are mostly used in hospitals. Therefore, low-cost bilateral hand training in a living environment is necessary.

## 3. Hand Exoskeleton for Bilateral Hand Training

**Figure 1:**
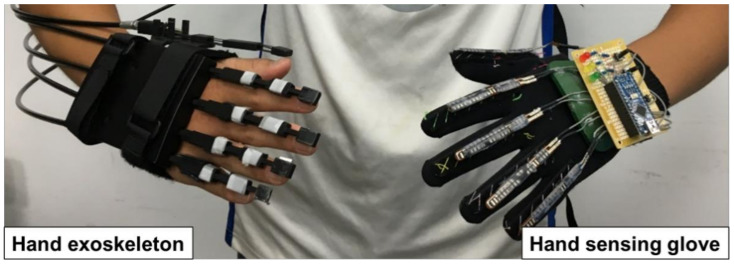
Framework of the bilateral hand practice, including a hand exoskeleton and a hand-sensing glove.

In this study, we designed a lightweight and portable hand exoskeleton with a hand-sensing glove for bilateral hand training, as shown in Figure 1. This system benefits patients with unilateral hemiplegia, but it can control the muscles of the unilateral hand. The control command was measured from the hand-sensing glove on the less-affected hand. Thereafter, the affected hand was then driven by the hand exoskeleton using a control command. This facilitated the movement of the affected hand by the less-affected hand. Bilateral hand training using a hand exoskeleton and hand-sensing glove promotes home-based rehabilitation for patients with unilateral hemiplegia. Furthermore, a virtual reality game was designed to increase the willingness of patients to perform bilateral hand training. The virtual reality game allows users to interact with objects and provides force feedback from the hand exoskeleton, maximizing patients’ opportunity for recovery. The demonstration video can be found at https://www.youtube.com/watch?v=S4n6x-JxGG8 (accessed on 9 September 2021).

### 3.1. Design of Hand Exoskeleton

**Figure 2:**
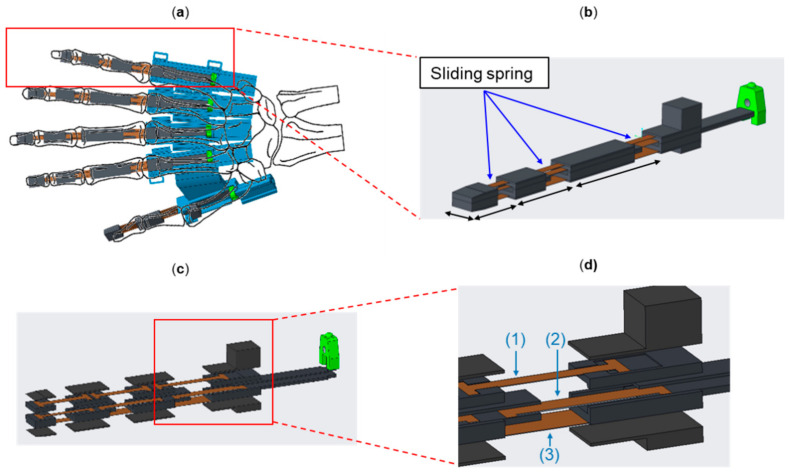
Hand exoskeleton (**a**) overview of the hand exoskeleton, (**b**) single-finger skeleton, (**c**) exploded view of the sliding spring, and (**d**) passive, active, and fixed sliding springs which are indicated by (1), (2), and (3), respectively.

We designed the hand exoskeleton motivated by Nycz et al. [12]. Different from their hand exoskeleton, we designed a thumb mechanism, enabling pinch or grasp gesture required for daily activities. The thumb and other four finger mechanisms can be actuated independently. Therefore, the degree of freedom of the hand exoskeleton is five. The preliminary mechanism of the hand exoskeleton is designed as shown in Figure 2a. Each finger exoskeleton is driven by a flexible sliding spring (JIS G3311, SK85M, Young’s modulus is 201–216 GPa) in brown color. Figure 2b shows a single-finger exoskeleton. This single-finger exoskeleton consists of three joints and is adopted for the index, middle, ring, and little fingers. The length of each finger exoskeleton differs from each other. The lengths of five fingers can be customized according to the user’s fingers. A single-finger exoskeleton with two joints is designed for the thumb mechanism. The single-finger exoskeleton performs flexion or extension by pushing or pulling the sliding spring, respectively.

**Figure 3:**
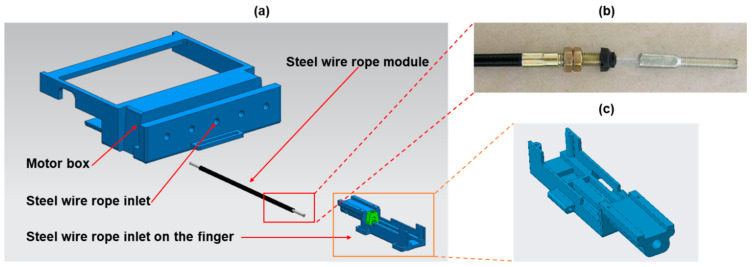
Transmission mechanism (**a**) assembly drawing, (**b**) Bowden cable module, and (**c**) connecting mechanism.

The single-finger exoskeleton consists of three layers of sliding spring, as shown in Figure 2c,d. The top, middle, and bottom layers represent passive, active, and fixed sliding springs, respectively. The active sliding spring bends when a push force is exerted on the green box. The passive sliding spring passively bends according to the movement of the active sliding spring. The force exerted on the green box is generated by a DC linear motor (Brushed DC Motor Linear Actuator, L12-30-100-6-P, Torque 151.07 oz-in) and is transmitted via a Bowden cable whose ends were soldered onto a *M3* solder extender followed by a *M3* Clevis, as shown in Figure 3. The Bowden cable transmits the force from the DC linear motor (motor box) to the finger mechanism, as shown in Figure 3a. The use of the Bowden cable allows the actuator (DC linear motor) to be equipped at the far end (at the user’s back or on the wheelchair). This reduces the loading of the hand and further makes the hand exoskeleton lightweight. The frame of the hand exoskeleton is made by 3D printing with acrylonitrile butadiene styrene.

The Bowden cable could only transmit approximately 10 N force. However, it is useful for subacute stroke patients with unilateral hemiplegia. This hand exoskeleton may not be useful for chronic stroke patients who have strong muscle spasticity because they need larger driving force. Only the proximal interphalangeal (PIP) and metacarpophalangeal (MCP) joints are actuated by the hand exoskeleton. For the index, middle, ring, and little fingers, the maximum flexion angles of PIP and MCP joints are 25 and 40 degrees, respectively. For the thumb, the maximum flexion angles of PIP and MCP joints are 30 and 40 degrees, respectively. The hand exoskeleton enables proportional control of the flexion angle for each finger, i.e., the flexion angle is proportion to the flexion extension degree of each finger measured in the less-affected hand. Details of measuring flexion-extension degree of each finger are provided in Section 3.4.

**Figure 4:**
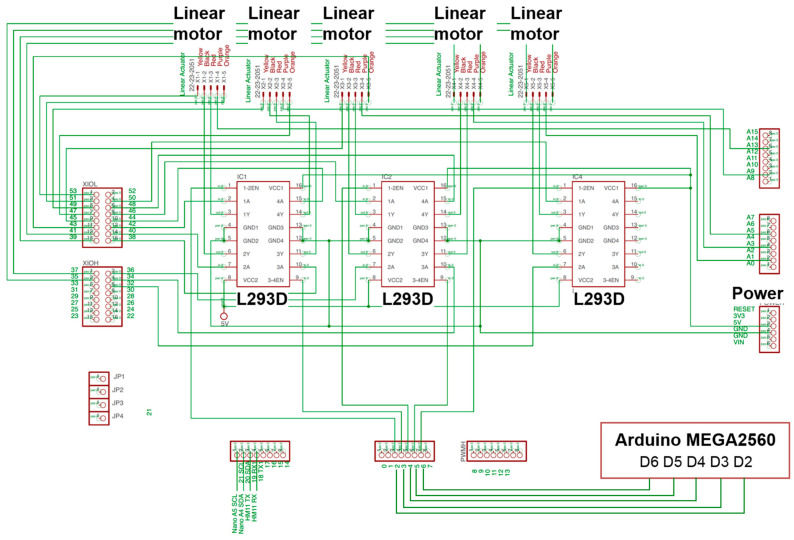
Electronic circuit of hand exoskeleton.

Figure 4 shows the electronic circuit of the hand exoskeleton. Each linear motor is connected to a Dual H-Bridge L293D motor driver. A microcontroller (Arduino MEGA2560) generates PWM signal to the motor driver. Total mass of the complete system is 1040 g, where 150 g is associated with the hand exoskeleton, 850 g is associated with the linear motors and battery, and 40 g is associated with the hand-sensing glove. The complete system is powered by a 6 V Li-Polymer battery with a capacity of 20,000 mAh. The system could continuously perform finger flexion and extension movements for 2 h.

### 3.2. Design of Thumb Mechanism

The thumb mechanism is designed to perform the pinch gesture for activities of daily living, as shown in Figure 2a in Section 3.1. We fixed the thumb mechanism adjacent to the index finger. This mechanism does not allow for relative movement between the thumb and index mechanisms. Hand motor impairment after stroke typically results in abnormal relative movements. Therefore, we fixed the thumb mechanism instead of making it free. The thumb mechanism performs flexion or extension by pushing or pulling the sliding spring indirectly, which is by a DC linear motor. Notably, the thumb mechanism has only two joints with identical actuation to those of the other four fingers.

### 3.3. Accurate Measurement of Displacement of the Sliding Spring

**Figure 5:**
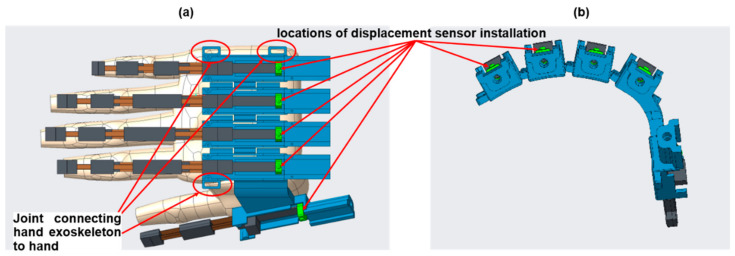
(**a**) Top view of the displacement sensor installation on the hand exoskeleton, (**b**) front view of the displacement sensor installation.

We equipped a force-sensing linear potentiometer (105 mm × 12 mm, Interlink Electronics, Irvine, CA, USA) to measure the translational displacement of the sliding spring. The measured displacement is adopted as a feedback signal to the controller to generate the control command. The displacement sensor is installed in the green box, which receives force from the Bowden cable and applies force to the sliding spring, as shown in Figure 5. Although the linear motor has a sensor to measure the applied displacement, the Bowden cable may deform during movement due to its mechanical property, resulting in inaccurate displacement on the hand side, i.e., inaccurate displacement of the sliding spring. Therefore, the displacement sensor installed at the joint between the sliding spring and the Bowden cable could provide accurate measurement to the controller for better actuation.

The hands perform various gestures for activities of daily living, resulting in various hand shapes. Anatomy suggests that the relative rotary movement between the fingers is in the range of 0–20 degrees; therefore, the fingers cannot be located in the same plane. A hand exoskeleton designed by Nycz et al. [12] provides a fixed shape for the back of the hand. This design is useful when a user performs an identical gesture. However, the shape of the back of the hand changes when the user performs various gestures. To achieve a comfortable experience for activities of daily living, we designed a flexible mechanism for the back of the hand such that the hand exoskeleton could be flexibly attached to the hand, as shown in Figure 5. The flexible mechanism is achieved by a passive joint between two finger exoskeletons. The flexible mechanism could be adaptively attached to the back of the hand, making the experience more comfortable and reducing restriction of the hand functions. An elastic band fixes the exoskeleton to the hand and it is fastened to three joints, as shown in Figure 5a.

Comparing our hand exoskeleton with that designed by Nycz et al. [12], we pursued two main improvements: (1) a flexible mechanism for the back of the hand and (2) a thumb mechanism. The flexible mechanism allows the hand exoskeleton to adaptively fit the shape of the back of the hand, improving the wearing experience and natural movement. The thumb mechanism makes the pinch gesture possible.

### 3.4. Design of the Hand-Sensing Glove

**Figure 6:**
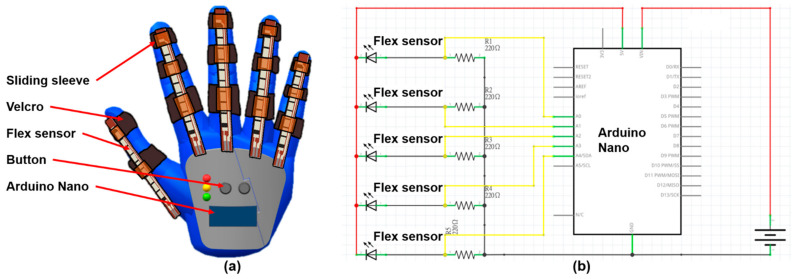
(**a**) Hand-sensing glove; (**b**) electronic circuits of the hand-sensing glove.

Bilateral hand training guides the affected hand to imitate the movement of the less-affected hand. We designed a hand-sensing glove to measure the movement of the less-affected hand, as shown in Figure 6a. The measured movement was adopted as the control command of the hand exoskeleton equipped on the affected hand. The flex sensor (Product No. FS2-L-055-103-ST, Spectra Symbol, Salt Lake, UT, USA) is equipped on each finger to measure the bending degree of each finger motion in the range from 0 to 120 degree. A microprocessor, i.e., the ATmega328 on the Arduino Nano evaluation board (Product No. A000005, Arduino, Somerville, MA, USA), converts the analog signal acquired from the flex sensor into digital signal with 10-bit resolution, which is further transmitted to the hand exoskeleton. The button allows the user to initialize the hand-sensing glove. Figure 6b shows the configuration of the electronic circuits of the hand-sensing glove.

### 3.5. Virtual Reality Game

Virtual reality games aim to increase the willingness of patients with unilateral hemiplegia to undergo rehabilitation. People with unilateral hemiplegia should perform repeated movement practice more than 300 times per training session to drive optimal neural reorganization [9]. However, the typical amount of repeated movement practice provided in current clinical settings is only 32 times on average, which is significantly less than the adequate amount. Therefore, we designed a virtual reality game to increase the amount of movement practice for home-based rehabilitation.

**Figure 7:**
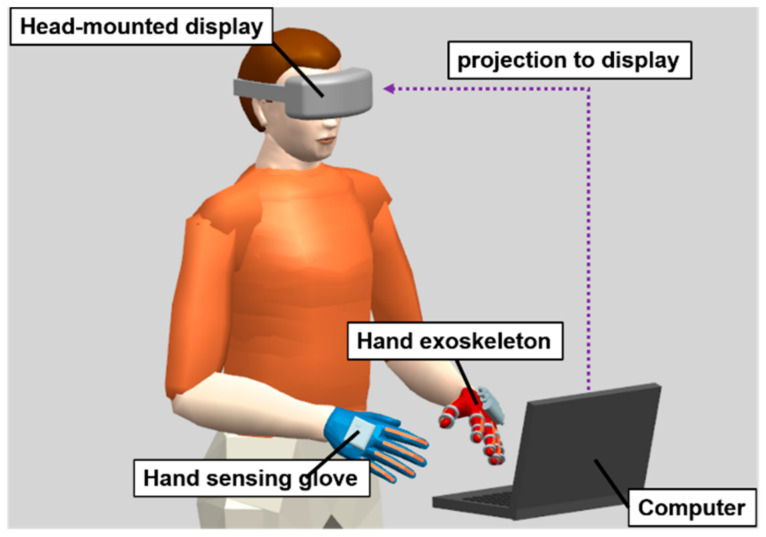
Framework of the virtual reality game including hand exoskeleton, hand-sensing glove, computer, and head-mounted display (optional).

**Figure 8:**
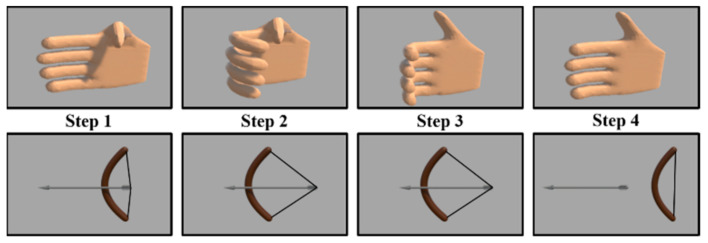
Schematic of virtual archery game. The user intends to control the virtual hand by the less-affected hand of the user.

The virtual reality game consists of four components: hand exoskeleton, hand-sensing glove, computer, and display, as shown in Figure 7. The user performs virtual archery by controlling the virtual hand to pull the bow, as shown in Figure 8. As the user performs an archery gesture by the less-affected hand, the hand-sensing glove measures the flexion or extension, and transmits the signal to the computer. The virtual hand is controlled by the hand-sensing glove and immediately actuates the bow to launch an arrow. A proportional control is applied to the virtual hand where the flexion extension degree measured by the hand-sensing glove is the input and the flexion extension degree of the virtual hand is the output. The flexion extension degrees of the less-affected hand are averaged over the index, middle, ring, and little fingers. The proportional controller receives the averaged flexion extension degree as the input and determines the output by multiplying the input by a gain for the virtual hand. The index, middle, ring, and little fingers perform an identical flexion extension according to the control output. The maximum flexion angles of PIP and MCP joints of the virtual hand are 70 and 60 degrees, respectively. Note that the maximum flexion angles of the virtual hand are larger than those of the hand exoskeleton. For the thumb of the virtual hand, the maximum flexion angles of PIP and MCP joints of the virtual hand are 65 and 70 degrees, respectively.

The hand exoskeleton is actuated by the hand-sensing glove to perform the bilateral hand training. The user obtains visual feedback from either a computer monitor or a head-mounted display to ensure whether the arrow lands in the correct position. For a computer monitor, a viewing distance of 65 cm, suggested in [29], is adopted. For a head-mounted display, a smart mobile phone (Butterfly, HTC Corporation, Taiwan) inserted into a VR Box (CeoMate Technology Co., Ltd., New Taipei City, Taiwan) is adopted. The smart mobile phone provides a high resolution (1080 p) and large display size (5 inches). The image of the virtual reality game in the computer is transmitted to the screen of the smart mobile phone using Bluetooth. The head movement is measured by the smart mobile phone and is transmitted to the computer for controlling the first-person perspective powered by Unity 3D game engine. A head-mounted display could increase a 3D game’s immersion, but it may cause temporary discomfort [30] for long-term use. Therefore, the head-mounted display is optional. The virtual archery game provides an alternative way to perform bilateral hand training, increasing the user’s willingness to consecutively perform repeated movement practice for better neural reorganization. People with unilateral hemiplegia can be motivated to undergo rehabilitation by the virtual reality game.

The virtual archery game consists of four steps, as shown in Figure 8. The user bends the thumb to fill the bow with an arrow when there is no arrow initially in the first step. The second step determines the degree of bow pulling according to the measured bending degree of the hand-sensing glove. The third step locks the bow, and the user straightens the thumb for a while. Finally, the user straightens four fingers to launch an arrow. The display from either a computer or head-mounted display shows the movement of the virtual hand and the trajectory of the arrow. The greater the bend, the tighter the bowstring is pulled, and the arrow can go farther. This helps the patient to observe the trajectory of the arrow better.

## 4. Implementation

### 4.1. Hand-Sensing Glove

**Figure 9:**
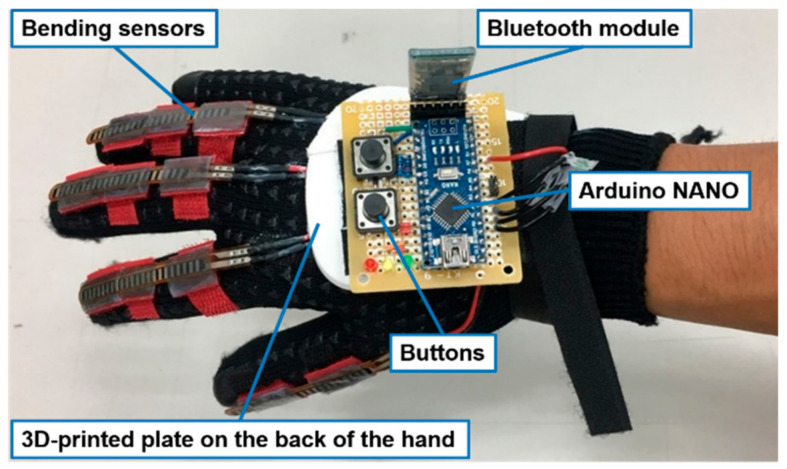
Implemented hand-sensing glove.

We implemented the hand-sensing glove as shown in Figure 9. The Arduino Nano evaluation board was adopted as the microprocessor to convert the bending signal into a digital signal. The control command was transmitted by a Bluetooth module. A plate on the back of the hand was manufactured by a 3D printing technique (3D printing with acrylonitrile butadiene styrene). Because the Arduino Nano evaluation board is lightweight, it was mounted on the back of the hand to prevent transmission noise from the long wire. Furthermore, signal transmission through the Bluetooth module reduces the inconvenience caused by the initial wire installation when the user starts the bilateral hand training.

### 4.2. Virtual Archery Game

**Figure 10:**
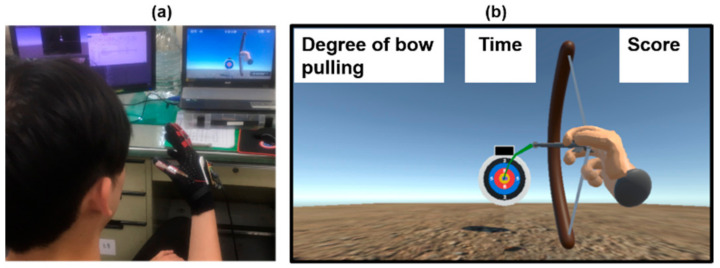
Demonstration of the virtual archery game. (**a**) The user is playing the virtual archery game. (**b**) The bow is pulled.

We implemented the virtual reality game using the Unity 3D game engine. When the user fills the bow with an arrow using the less-affected hand, the user pulls the bow by bending four fingers, as shown in Figure 10. The affected hand is bent by the hand exoskeleton with identical control command to that of the virtual hand. When the arrow lands on the target, the user obtains a score. The user obtains 10 points for hitting the yellow center, 8 points for the red area, 5 points for the blue area, 2 points for the black area, and 0 points for the rest.

### 4.3. Bilateral Hand Training

**Figure 11:**
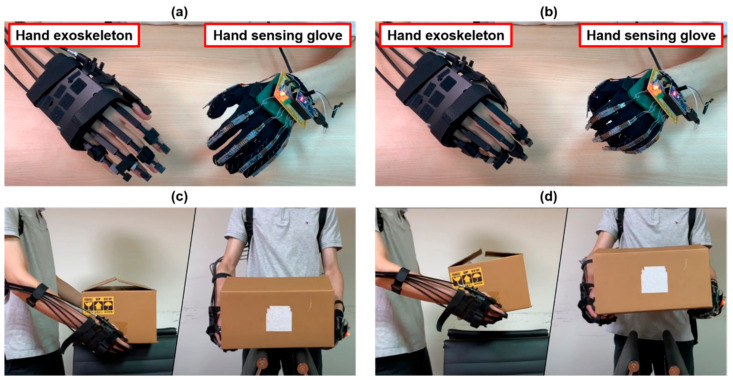
Bilateral hand training (**a**,**b**) and application of the hand exoskeleton for activities of daily living (**c**,**d**).

We implemented the hand exoskeleton for bilateral hand training, as shown in Figure 11a. The hand exoskeleton is controlled by a hand-sensing glove that then actuates the affected hand. However, when the less-affected hand exhibits a large bending degree, the hand exoskeleton exhibits only a small bending degree, as shown in Figure 11b. The bending degree of the hand exoskeleton depends on the length of the sliding spring. That is, the longer the sliding spring, the greater the bending degree. The user cannot squeeze the fist due to the length of the user’s finger. However, this does not affect bilateral hand training because the rehabilitation for people with unilateral hemiplegia typically involves incremental training. The somatosensory and motor feedback provided to the affected hand by the guidance of the hand exoskeleton during bilateral hand training may still facilitate neural reorganization for their hand motor control. Additionally, the hand exoskeleton assists in activities of daily living possible. Figure 11c,d show that the hand exoskeleton facilitates the flexion of the affected hand; thus, the user could lift a lightweight box using two hands.

## 5. Conclusions

We propose a hand exoskeleton and a hand-sensing glove for bilateral hand training to restore hand function in people with unilateral hemiplegia. The hand exoskeleton is actuated by a sliding spring, and its actuator could be equipped at the far end to reduce the loading of the hand. Compared with the existing hand exoskeletons, the flexible mechanism for the back of the hand has been improved and the thumb mechanism is designed to make the pinch gesture possible. The hand-sensing glove measures the bending degree of the less-affected hand by a flex sensor and generates a control command for the affected hand. A virtual reality game is designed to encourage people to perform a large amount of repeated and correct movement practice. The system could optimize the recovery of hand function for people with hemiplegia by sufficient bilateral hand training. Future works will consider various virtual reality games for various rehabilitation therapies.

## Data Availability

Not applicable.
